# Supplementary material for: MUC1 Expressions and Its Prognostic Values in US Gastric Cancer Patients
Source: Cancers (Basel). 2023 Feb 4;15(4):998. doi: 10.3390/cancers15040998 (PMC9954699; doi:10.3390/cancers15040998)
Supplement: Supplementary file 1 [file cancers-15-00998-s001.zip › cancers-2134001-supplementary.pdf]

Supplementary Tables

Table S1. Mucin expressions and pathological characteristics of gastric cancer

	MUC2			MUC5AC*			MUC6*		
	Negative (no= 15)	Positive (no= 55)	*p*	Negative (no= 17)	Positive (no= 52)	*p*	Negative (no= 46)	Positive (no= 23)	*p*
Tumor size (cm), mean±SD	3.9±2.4	3.6±2.4	0.669	3.6±2.4	3.7±2.4	0.886	3.7±2.2	3.7±2.8	0.890
Tumor location, no (%)			0.265			0.083			0.354
Upper third	6 (40.0)	32 (58.2)		11 (64.7)	27 (51.9)		26 (56.5)	12 (52.2)	
Middle third	3 (20.0)	12 (21.8)		5 (29.4)	9 (17.3)		11 (23.9)	3 (13.0)	
Lower third	6 (40.0)	11 (20.0)		1 (5.9)	16 (30.8)		9 (19.6)	8 (34.8)	
Background intestinal metaplasia, no (%)			0.130			0.732			0.606
Absent	9 (60.0)	21 (38.2)		8 (47.1)	22 (42.3)		21 (45.7)	9 (39.1)	
Present	6 (40.0)	34 (61.8)		9 (52.9)	30 (57.7)		25 (54.3)	14 (60.9)	
Lauren classification, no (%)			0.885			0.619			0.103
Intestinal type	11 (73.3)	35 (63.6)		12 (70.6)	33 (63.5)		26 (56.5)	19 (82.6)	
Diffuse type	4 (26.7)	17 (30.9)		4 (23.5)	17 (32.7)		17 (37.0)	4 (17.4)	
Mixed type	0 (0)	3 (5.5)		1 (5.9)	2 (3.8)		3 (6.5)	0 (0)	
Tumor depth, no (%)			0.071			0.344			0.351
Mucosa	0 (0)	11 (20.0)		1 (5.9)	9 (17.3)		5 (10.9)	5 (21.7)	
Submucosa	5 (33.3)	8 (14.5)		5 (29.4)	8 (15.4)		8 (17.4)	5 (21.7)	
Proper muscle or deeper	10 (66.7)	36 (65.5)		11 (64.7)	35 (67.3)		33 (71.7)	13 (56.5)	
Perineural invasion, no (%)			0.796			0.732			0.482
Absent	9 (60.0)	35 (63.6)		10 (58.8)	33 (63.5)		30 (65.2)	13 (56.5)	
Present	6 (40.0)	20 (36.4)		7 (41.2)	19 (36.5)		16 (34.8)	10 (43.5)	

Lymphovascular invasion, no (%)			0.073			0.948			0.495
Absent	4 (26.7)	29 (52.7)		8 (47.1)	24 (46.2)		20 (43.5)	12 (52.2)	
Present	11 (73.3)	26 (47.3)		9 (52.9)	28 (53.8)		26 (56.5)	11 (47.8)	
Lymph node metastasis, no (%)			0.041			0.728			0.395
Absent	4 (26.7)	31 (56.4)		9 (52.9)	25 (48.1)		21 (45.7)	13 (56.5)	
Present	11 (73.3)	24 (43.6)		8 (47.1)	27 (51.9)		25 (54.3)	10 (43.5)	
Distant metastasis, no (%)			0.109			0.441			0.323
Absent	9 (60.0)	44 (80.0)		14 (82.4)	38 (73.1)		33 (71.7)	19 (82.6)	
Present	6 (40.0)	11 (20.0)		3 (17.6)	14 (26.9)		13 (28.3)	4 (17.4)	
AJCC TNM stage, no (%)			0.504			0.943			0.340
Stage I	4 (26.7)	20 (36.4)		5 (29.4)	18 (34.6)		12 (26.1)	11 (47.8)	
Stage II	3 (20.0)	13 (23.6)		5 (29.4)	11 (21.2)		12 (26.1)	4 (17.4)	
Stage III	2 (13.3)	11 (20.0)		3 (17.6)	10 (19.2)		9 (19.6)	4 (17.4)	
Stage IV	6 (40.0)	11 (20.0)		4 (23.5)	13 (25.0)		13 (28.3)	4 (17.4)	

Abbreviations: MUC, mucin; SD, standard deviation; AJCC, American Joint Committee on Cancer.

*One patient did not have results of immunohistochemical stain for MUC5AC and MUC6.

Table S2. MUC1 expression and pathological characteristics of surgically treated gastric cancer without distant metastasis

	MUC1		p
	Negative (no= 18)	Positive (no= 31)	
Tumor size (cm), mean±SD	3.1±2.0	4.4±2.8	0.095
Tumor location, no (%)			
Upper third	3 (16.7)	4 (12.9)	0.706
Middle third	6 (33.3)	8 (25.8)	
Lower third	9 (50.0)	19 (61.3)	
Background intestinal metaplasia, no (%)			
Absent	4 (22.2)	15 (48.4)	0.070
Present	14 (77.8)	16 (51.6)	
Lauren classification, no (%)			
Intestinal type	18 (100)	16 (51.6)	0.001
Diffuse type	0 (0)	12 (38.7)	
Mixed type	0 (0)	3 (9.7)	
Tumor depth, no (%)			
Mucosa	7 (38.9)	2 (6.7)	0.002
Submucosa	6 (33.3)	5 (16.7)	
Proper muscle or deeper	5 (27.8)	23 (76.7)	
Perineural invasion, no (%)			
Absent	15 (83.3)	18 (58.1)	0.069
Present	3 (16.7)	13 (41.9)	
Lymphovascular invasion, no (%)			
Absent	12 (66.7)	12 (38.7)	0.059
Present	6 (33.3)	19 (61.3)	
Lymph node metastasis, no (%)			
Absent	14 (82.4)	13 (46.4)	0.017
Present	3 (17.6)	15 (53.6)	
AJCC TNM stage,* no (%)			
Stage I	13 (72.2)	7 (22.6)	0.002
Stage II	4 (22.2)	12 (38.7)	
Stage III	1 (5.6)	12 (38.7)	

Abbreviations: MUC, mucin; SD, standard deviation; AJCC, American Joint Committee on Cancer.

*Final pathological cancer stages were classified according to the 7th edition of AJCC TNM staging classification.
